# Variability of expert assessments of ECG time domain parameters

**DOI:** 10.1097/EA9.0000000000000020

**Published:** 2023-02-17

**Authors:** Carl Böck, Christoph Mörtl, Christoph Mahringer, Mario Huemer, Jens Meier

**Affiliations:** From the JKU LIT SAL eSPML Lab, Institute of Signal Processing, Johannes Kepler University Linz, Linz, Austria (CB, MH), University Clinic of Anesthesiology and Critical Care Medicine, Johannes Kepler University Linz, Linz, Austria (CMö, JM), Department for Medical Engineering, Kepler University Hospital, Linz, Austria (CM)

## Abstract

**BACKGROUND:**

In anaesthesiology, time domain parameters of the ECG are frequently used for long QT diagnosis, determination of branch blocks and for identifying atrioventricular blocks. However, this analysis depends on several factors and therefore the time domain parameters are prone to variability.

**OBJECTIVE:**

To determine the variability of expert assessments of ECG time domain parameters in daily clinical practice.

**DESIGN:**

In an observational study 18 physicians labelled the onset, peak and end of the waves (P-QRS-T) for 26 preselected, distinctive beats. Based on this, clinically important durations and intervals were derived: the duration of the P wave, T wave and QRS complex, as well as the length of the PQ and QT interval. These parameters were subsequently analysed with respect to inter-expert variability (for all experts and discipline-based subgroups) and, additionally, were compared with computer-aided analysis.

**SETTING:**

ECG recordings from participating patients were recorded in March 2015 during standard examination in the hospital and stored in the local ECG database.

**PATIENTS:**

We did not define inclusion or exclusion criteria for the patients themselves; the ECG beats were selected by a medical expert with respect to their shapes and abnormalities.

**MAIN OUTCOME MEASURES:**

Variability of clinically relevant ECG wave durations and intervals quantified were expressed as the interquartile range and the Q_2_/Q_98_ span for 18 investigators.

**RESULTS:**

The resulting wave durations (P_dur_, QRS_dur_ and T_dur_) and intervals (PQ and QT) showed high variability, for instance, captured by the Q_2_/Q_98_ span ranging from 39 to 99 ms.

**CONCLUSION:**

The observed, inter-investigator variability of assessing the PQ and QT intervals, as well as the wave durations, might result in important variance regarding ECG-associated diagnoses. Whether these variances play a major role in typical clinical situations would have to be demonstrated with further clinical observational studies.

**KEY POINTS**

- ECG wave durations and intervals have been used as nonspecific screening tools and are therefore an important cornerstone in diagnostic decision making.
- ECG wave boundaries are frequently difficult to determine, affecting the reliability of the derived parameters.
- We quantified the variability of clinically relevant time domain parameters.
- The observed variability of ECG time domain parameter assessments is generally rather high and might influence the treatment path of patients as well as the performance measures of computer-aided ECG delineation.

## Introduction

ECG analysis is a cornerstone of many cardiological diagnoses and treatment pathways and is often used as a nonspecific screening tool.^1^ There is a large range of diseases and pathophysiology which are mainly diagnosed by various features of the ECG that either have been analysed manually or automatically by an algorithm embedded in an ECG recorder. Very often these analyses result in invasive investigations, e.g., percutaneous coronary procedures^2^ or the necessity to start lifelong medications.^3^ The ECG features used to signify different conditions are frequently determined by the morphology of the ECG trace (e.g. ST segment changes) or depend on the length of specific waveforms or on the separation between them. Important examples for the latter are ECG changes that signify bundle branch blocks, atrioventricular blocks or long QT syndromes. For example, the QT interval represents the duration of ventricular depolarisation and subsequent repolarisation. It is measured from the beginning of the QRS complex to the end of the T wave. The onset of the Q wave and the end of the T wave are defined by the earliest onset of a Q wave and the end of the last T wave in one lead.^4^ Although modern signal processing algorithms have the potential to measure these time intervals automatically, at present, technicians or physicians still often determine them manually.

Both approaches share the same problem: the beginning and the end of the QT interval are not defined by sharp deflections of the ECG trace but by gentle ascents and descents.^5^ This specific waveform leaves room for interpretation, and consequently, it is difficult to determine the most precise duration of the QT interval. The same might hold true for the P wave and, consequently, for the PQ interval. Therefore, the determination of these intervals seems to be open to subjective interpretation by the investigator. Given these inaccuracies, one can easily imagine that diagnoses based on these features may be imprecise. Surprisingly, to date, there is not a single study that has systematically investigated this potential inaccuracy of ECG interval determination. Since most computer algorithms are tested against human experts (considered being the gold standard), the assessment of their performance may depend essentially on the interpretations of these human experts.

It is, therefore, the aim of our study to describe inter-investigator variability for different ECG waves and intervals, to describe potential influencing factors in interpretation. We hypothesise that, depending on the actual morphology of the ECG, there may be clinically relevant variability.

## Material and methods

### Selection of ECG samples

**Fig. 1:**
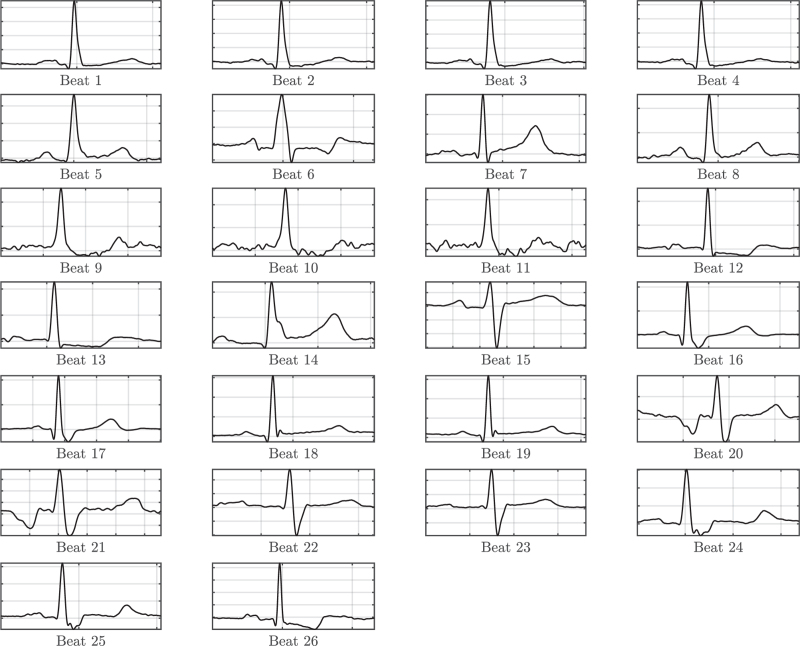
Set of 26 ECG beats selected for investigating the variability of the expert assessments.

Ethical approval for our study (I-12-14) was provided by the Ethics Committee of Upper Austria (Ethikkommission des Landes Oberösterreich, Linz, Austria, Chairperson Prof Dr Johannes Fischer) on 3 November 2014. In March 2015, standard three-lead ECGs were recorded using a digital recorder (Schiller Medilog AR12) with a digital time resolution of 1 ms and stored in the ECG database of the Kepler University Hospital. Subsequently, a senior physician selected 26 representative ECG beats from this database. Each of the 26 beats chosen differed from the others in terms of morphology and time intervals, and the actual selection of each beat was carried out by one medical expert (CMö). Every beat was presented once to the investigators to exclude influences by repetitive analysis of the signal. An overview of the single beats is given in Fig. 1.

### Investigators

Only investigators with experience in ECG analysis were invited to participate. A further inclusion criterion was the expert's availability to analyse all of the beats presented within a single session. Finally, 18 investigators were included, of whom seven were anaesthesiologists, five were intensivists and six were internal medicine physicians. Each physician received detailed information at the beginning of the study about the operating principle of the computer software used for ECG labelling. If a participant could not completely analyse the whole data set of the ECG beats being studied, their analysis was omitted and they were substituted by another colleague.

### Determination of ECG time points

To allow efficient and fast labelling by the clinicians, a MATLAB graphical user interface for annotating the onset, peak and end of P, Q, R, S, and T waves was implemented. This software consisted of a window where each beat was depicted, and the distinct time points could then be labelled with different markers. The markers could be altered throughout the analysis allowing investigators to modify their choices as necessary. The MATLAB setup provided an upper limit of annotating 11 labels per beat. Furthermore, features like the possibility of labelling waves as ‘not present’, an automatic zoomed view or the possibility to scroll through the ECG sequence were implemented as well but are not discussed in detail here. The ECGs were presented to each participant in the same manner: The beats were depicted one after the other, and the experts were then asked to label the following characteristic time points: onset, peak and end of the P wave (P_on_, P_peak_, P_end_), onset and end of the QRS complex (QRS_on_, QRS_end_) as well as the peaks of the Q, R and S waves (Q_peak_, R_peak_, S_peak_), and onset, peak and end of the T wave (T_on_, T_peak_, T_end_). Thereafter, the duration of the P wave, the QRS complex and the T wave, as well as the PQ interval and the QT interval were calculated and stored automatically for further data analysis. The experts were blinded for their individual results and the results of the other investigators.

### Statistical analysis

Clearly, due to the use of real-life data, there is no objective gold standard of the true onset, peak or end of a specific wave. Even a highly experienced cardiologist or any sophisticated computer algorithm might be prone to bias. Consequently, we decided to use the median of the experts as a gold standard. Thereafter, for each time point, descriptive statistics were determined as follows: for every beat separately, the median of the expert assessments was calculated for each label, leading to 11 ‘true’ labels per ECG beat for the features of interest. Subsequently, the difference of each expert's label from the ‘true’ value was determined. To estimate the variance of these differences – corresponding to the spread of the expert labels themselves – the interquartile range (IQR) and the Q_2_/Q_98_ span were calculated for every beat separately, as well as for the whole dataset.

Furthermore, and clinically even more relevant, the distributions of the resulting wave durations (P_dur_, QRS_dur_, T_dur_) and intervals (PQ, QT) were captured by determining the median, IQR, Q_10_/Q_90_ span, Q_2_/Q_98_ span as well as the total range for every beat and subsequently averaged over all beats. Hence, the main outcome parameters of our study are considered to be the averaged IQR and Q_2_/Q_98_ span of these durations and intervals, robustly reflecting inter-investigator variability and its possible impact on ECG-associated diagnoses. Subsequently, we also analysed the results regarding any differences between the three medical specialties of anaesthesiology, intensive care medicine and internal medicine. For that, we determined the average median, IQR and Q_2_/Q_98_ span for these three professional groups separately and presented the results in a descriptive table.

In addition, as computer-aided parameter detection has become the standard for interpreting telemetry strips in daily clinical practice, we also analysed the differences between the algorithm assessments and the expert labels, the latter again serving as gold standard. We determined the median deviations (bias) of the algorithm labels from the single experts as well as the IQRs (scatter) of the expert-dependent differences. Consequently, we obtained performance measures of the algorithm for every single expert and could explore the differences from expert to expert. The respective wave durations and intervals were determined by the well-known wavelet-based ECG delineation algorithm proposed by Martinez *et al.*^6^

## Results

**Fig. 2:**
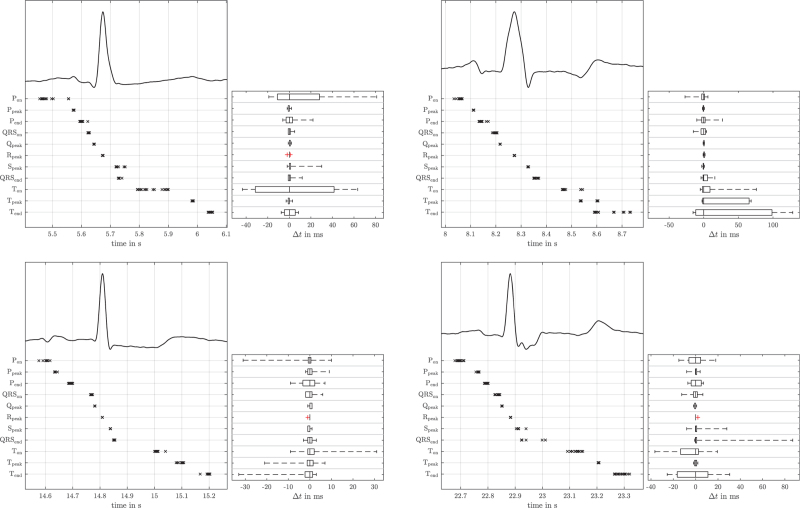
Example beats with expert labels below the ECG trace and the according boxplots for the difference between the single expert labels to the median of the expert labels.

**Fig. 3:**
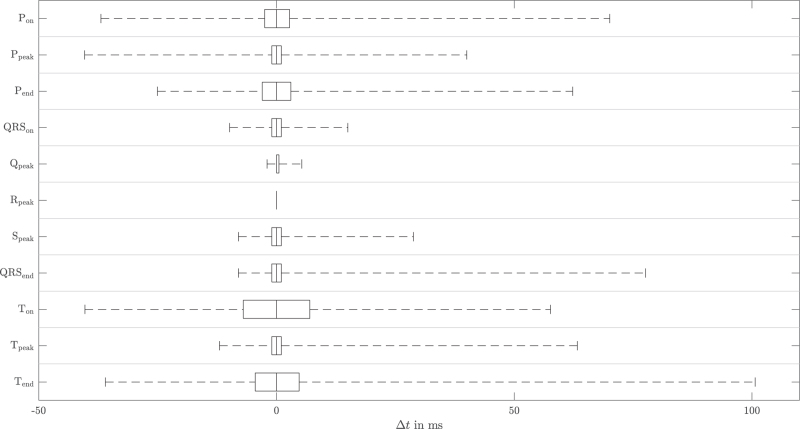
Differences between the expert labels and the median of the labels.

All 18 experts completed the whole protocol. This resulted in 468 fully annotated beats without any missing values. Figure 2 illustrates four example beats with their respective expert labels. The scattering of these labels is represented via boxplots showing the IQR and the 2nd/98th percentile, respectively. In Fig. 3, the differences between the labelled ECG characteristic points are summarised for the whole dataset. Again, the whiskers represent 96% of all expert labels (2nd/98th percentile). Hence, possible unintentional mislabelling by the experts is already allowed for by excluding the most extreme label differences in this illustration. Nevertheless, there were still seven points that were annotated with clinically relevant variability: P_on_, P_peak_, P_end_, QRS_end_, T_on_, T_peak_ and T_end_. Notably, the span between the 98th and the 2nd percentile ranged from 75 to 137 ms for these seven time points (Fig. 3). In addition, QRS_on_ and S_peak_ obviously resulted in relevant variability, although this variability was not as high as in the former seven time points.

Focusing on the derived clinically relevant parameters, notably wave durations and PQ/QT intervals, Table 1 captures the distribution by depicting the averaged median, IQR, Q_10_/Q_90_, Q_2_/Q_98,_ as well as the averaged total range. The calculated durations of the P wave, QRS complex and T wave resulted in IQRs of 16, 7 and 29 ms, respectively, whereas the IQRs for the PQ and the QT intervals were 19 and 15 ms, respectively. Analysing the Q_2_/Q_98_ span of these parameters, we noted that, in particular, the T wave duration and the QT interval seem to be difficult to determine. Table 2 illustrates the results for the specific professional groups; group 1 represents anaesthesiologists, group 2 intensive care specialists and group 3 summarises the results of the internists. In general, we did not observe major differences for the medians and the scatter between the different specialist groups. However, the difference between the Q_2_/Q_98_ span of group 1 (QRS duration, T wave duration, QT interval) and group 2/3 might indicate a variability across the professional groups. As the sample size of the subgroups was rather small, further investigations would be necessary to determine potential variability across specialties.

**Table 1:** Median, IQR, Q_90_ to Q_10_ span, Q_98_ to _Q2_ span and total range for the variability of expert assessments of wave durations as well as PQ and QT interval (averaged across 26 beats)

	Median	IQR	Q90 to Q10	Q_98_ to Q_2_	Max to Min
Pdur	97	16	33	53	59
QRSdur	117	7	25	39	43
Tdur	172	29	56	89	99
PQ interval	151	19	33	48	52
QT interval	403	15	39	68	74

All values are given in ms.

**Table 2:** Median, IQR and Q_90_ to Q_10_ span (averaged across 26 beats) separately analysed for each professional group (Group 1: anaesthesiology, Group 2: intensive care medicine and Group 3: internal medicine)

	Group 1			Group 2			Group 3		
	Median	IQR	Q_98_ to Q_2_	Median	IQR	Q_98_ to Q_2_	Median	IQR	Q_98_ to Q_2_
Pdur	95.6	12.1	33.2	94.2	15	33.5	101.1	12.5	33.5
QRSdur	118.3	6.1	32.1	116.6	9.4	18.7	117.2	8.8	20.5
Tdur	175	27	73.1	165.1	20.7	49.6	178.5	20.86	46.6
PQ interval	152.3	14.8	31.3	148.8	12.7	26.7	154.7	11	30.2
QT interval	405.9	14.6	52.6	401.4	16.29	29.3	402.7	17.2	40.8

All values are given in ms.

**Fig. 4:**
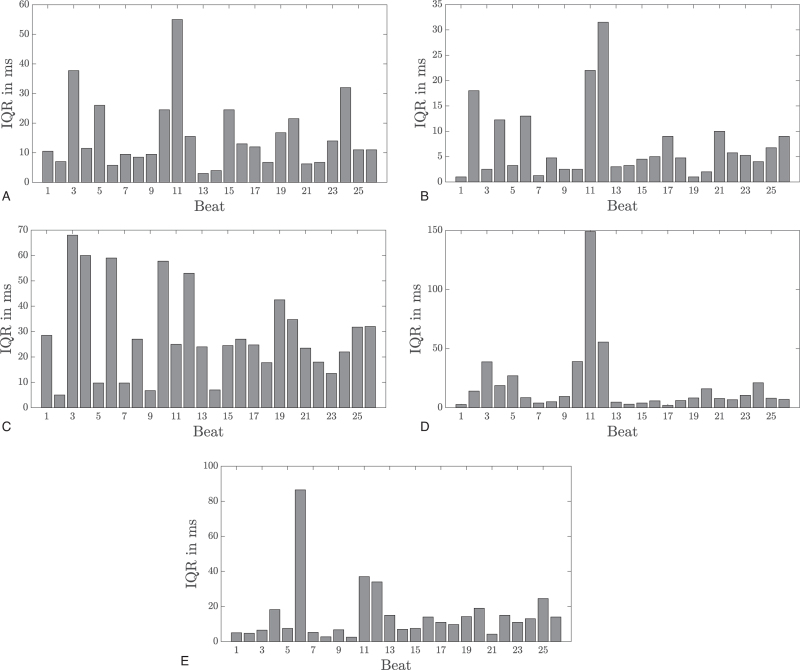
IQR of the difference between the expert labels and their medians (considered as ‘gold standard’) for all beats and different diagnostic durations/intervals. (a) P wave duration. (b) QRS complex duration. (c) T wave duration. (d) PQ interval. (e) QT interval.

Investigating the IQRs of the clinically relevant parameters for the single beats, a distinct heterogeneity was observable, depending on the individual beat (Fig. 4). For some specific beats, determination of the wave duration or an interval seems to be extremely difficult. For example, the IQR for the duration of the P wave was highest in beats 3, 11 and 24 (Fig. 4a), the IQR of the PQ interval was highest in beat 11 (Fig. 4d), and the variability in the QT interval was largest in beat 6 (Fig. 4e). Note that different physicians were responsible for the variation in these parameters. Depending on the individual shape of the ECG trace, IQRs of up to 150 ms could possibly influence clinical judgement of relevant differences when considering underlying diseases.

However, not only does the evaluation of underlying diseases constitute a major concern, but also the performance of computer-aided assessments needs to be questioned based on the comparison of human expert labels and algorithm labels. Regarding that, Table 3 shows, for every expert separately, the median deviations and IQRs between the expert and algorithm labels. The median may be interpreted as the bias between the algorithm and expert assessments, while the IQR reflects the scattering of the differences. We observed that the algorithm performance seemed to vary depending on the expert, illustrated by some significant differences for both metrics, the median and the IQR, where the biggest and the smallest values are highlighted in bold font in Table 3.

**Table 3:** Comparison between automated wave duration/interval determination and the determination by the single experts quantified by the median difference (between algorithm and expert), and the IQR capturing the scatter of the algorithm-expert differences. The biggest and smallest values for the median and IQR are highlighted in bold font for the wave durations/intervals investigated.

	P duration		QRS duration		T duration		PQ interval		QT interval	
	Median	IQR	Median	IQR	Median	IQR	Median	IQR	Median	IQR
Expert 1	−**1.00**	20.00	4.00	18.00	−8.00	54.00	−4.00	14.00	−2.50	15.00
Expert 2	−4.50	13.00	4.50	**14**.**00**	−**29**.**00**	57.00	−**8**.**00**	12.00	−8.00	**10**.**00**
Expert 3	−9.00	20.25	4.50	18.50	−18.00	59.25	−7.00	20.25	−3.00	20.50
Expert 4	2.00	19.00	4.50	18.00	−15.50	71.00	0.50	22.00	**0**.**00**	14.00
Expert 5	3.00	17.00	4.50	16.00	−23.00	53.00	−2.50	17.00	−2.00	16.00
Expert 6	−6.50	**11**.**00**	−2.00	21.75	19.50	**89**.**00**	−3.00	19.75	−2.00	24.75
Expert 7	2.00	15.00	11.00	27.50	−10.00	78.75	−5.00	22.50	0.50	17.50
Expert 8	−**13**.**00**	20.25	−9.00	**14**.**00**	−28.50	45.00	−3.00	**9**.**00**	−**10**.**00**	20.00
Expert 9	4.50	15.50	**1**.**50**	16.00	−18.50	50.00	0.50	16.00	−3.00	18.00
Expert 10	**1**.**00**	19.25	4.00	26.00	−25.50	45.00	−1.00	17.75	−5.50	28.00
Expert 11	2.50	23.50	**0**.**50**	18.00	−11.00	57.25	3.00	21.50	−1.00	24.25
Expert 12	−2.00	25.50	**21**.**50**	**53**.**50**	−17.50	49.50	1.00	25.50	−5.00	19.25
Expert 13	3.00	27.25	3.00	18.00	−15.00	79.00	4.00	**27**.**00**	−2.00	23.50
Expert 14	1.50	15.00	−**1**.**50**	16.00	−**6**.**00**	67.00	**0**.**00**	19.00	−6.00	17.00
Expert 15	5.00	16.50	4.00	17.00	−11.50	86.00	4.00	24.50	−3.50	18.00
Expert 16	1.50	22.00	2.00	23.00	−6.50	66.00	−2.50	19.00	−2.00	18.00
Expert 17	7.00	17.00	3.50	18.00	−11.00	53.00	**0**.**00**	17.00	−3.00	14.00
Expert 18	9.00	**37**.**50**	2.00	23.50	15.00	67.25	2.00	13.50	−1.00	**30**.**25**

All values are given in ms.

## Discussion and conclusion

The main result of our study was that the variability of assessments regarding several ECG time domain parameters is generally rather high and may thus influence the patient treatment pathways. ECG interpretation is essential knowledge acquired first at medical school, and its proper application is important for many different medical specialties.^7,8^ Furthermore, there is a large number of textbooks, tutorials and ‘how-tos’ that enable young physicians to work out a diagnosis from a 12-lead ECG.^8–10^ The consequence of this diagnosis may well be either an invasive procedure or lifelong medication, illustrating the need for accuracy.

In the last few years in particular, time domain parameters like the QT interval have attracted more and more attention. For example, long QT syndrome (LQTS), a condition that affects repolarisation of the heart after systole, has been associated with many different medications.^11^ The LQTS results in an increased risk of dysrhythmia, which can result in fainting, drowning or sudden death and, therefore, should be correctly diagnosed.^10^ For the determination of the QT interval, it is of utmost importance to capture the onset of the Q wave and the end of the T wave correctly.^12^ We observed an average Q_2_/Q_98_ span of 68 ms in our data, indicating that it is the determination of the end of the T wave that underlies certain variability. In other words, 96% of the investigators estimated the QT interval within a range of 68 ms. What seems to be theoretically acceptable on first viewing might play a huge role in daily clinical practice. In a recently published study by Rijnbeek *et al.*^13^ it was demonstrated that the typical Q_2_/Q_98_ span for the QT interval in healthy individuals over several ages is about 110 ms. A very similar result was found by Mason *et al.*^14^ and Christov *et al.*^15^ some years beforehand. We obtained a mean Q_2_/Q_98_ span of 68 ms, not for different patients, but for several measurements of the same beat by different investigators. Therefore, investigator-associated heterogeneity of QT measurement corresponds to more than 50% of naturally present patient-associated inter-individual heterogeneity. One might therefore speculate that the determination of the QT interval is quite difficult for the average physician and warrants special attention and training.^10,12^ Several reasons might be responsible for this phenomenon: the QT interval is different for different leads; an occasionally present U wave might influence the measurement of the end of the T wave; and the end of the T wave is actually difficult to determine.^12^ Although publications for the correct determination of the QT interval have existed for many years,^16^ valid and reliable measurement still seems difficult for most physicians and is prone to severe bias.

The situation was very similar for the P wave and the PQ interval in our study. We observed a mean inter-investigator Q_2_/Q_98_ span for the P wave duration of 53 ms and for the PQ interval of 48 ms. Again, this is very close to the natural inter-individual variability of healthy individuals, as demonstrated by Rijnbeek *et al*.^13^ In addition, this variability is rather high and might make correct diagnosis of various relevant conditions more difficult. In this study, the onset and the end of the P wave seemed difficult to determine. This is even more striking in cases like beat 11 in our examples, where a noisy signal makes the determination of these time points virtually impossible. Since it has been demonstrated that a short as well as a long P wave might be associated with atrial fibrillation,^17,18^ the variability described might also influence these measurements.

Surprisingly, the QRS duration was also quite difficult to determine. We observed an average Q_2_/Q_98_ span of the QRS duration of 39 ms in our investigation. Although the Q, the R and the S wave have relatively sharp edges, there are some cases, like in beats 11 and 24, where the specific wave boundaries could not be determined uniformly by all experts. The QRS duration is a very important predictor of myocardial hypertrophy and arrhythmia,^19^ and the results obtained would be important for the clinical management of a patient.

Consequently, one might state that the experts have not determined any of the typically measured intervals with high reliability or validity. This surprising finding questions the results of many ECG diagnoses and, as a consequence, many kinds of therapy. However, nowadays, most of these measurements are performed by computer algorithms. Nevertheless, one of the biggest challenges is the evaluation of ECG delineation algorithms, as there is no absolute ‘gold standard’ method of determining the time points for the ECG features studied. Hence, the performance of such delineation algorithms was assessed against the expert labelling, which was considered as ‘gold standard’. For instance, many algorithms were compared to the so-called Physionet QT database,^20^ where one expert labelled the wave limits and peaks of 105 distinct ECG recordings, comprising 3623 beats. Although this kind of evaluation provides a valid benchmark for comparison, one has to keep in mind that expert labels are prone to variability and, therefore, may strongly influence the performance of the algorithms.

This was illustrated in Table 3, where, depending on the expert, the delineation performance was strong, average or poor. For example, in the case of the QRS duration for expert 9, the algorithm achieved very good delineation results with a median deviation of 1.50 ms and an IQR of 16.00 ms. In contrast, the evaluation of the algorithm based on expert 12 revealed a median deviation of 21.50 ms and a broad deviation scatter (IQR 53.50 ms), which resembles very poor delineation performance.

The current issue should be mentioned when evaluating automated ECG algorithms based on human expert labelling, especially if there is no more than one expert, as was the case in Physionet.^20^ In general, we would suggest that computer-aided analysis has the advantage that the parameters described above are determined on a ‘blinded’ basis. However, all algorithms will have the disadvantage that they are essentially biased, as they can only detect ECG patterns that they have been programmed for. Any abnormality that has not been included during the development of the algorithm cannot be detected. The objectivity of computer algorithms is their main advantage. However, they are constrained regarding the number and type of conditions they can detect, and this is their greatest disadvantage.

Our study has several aspects that might weaken its findings. First, the selection of ECG beats themselves and their number was rather arbitrary, based on a broad variety of morphologies in the study database of the Kepler University Hospital. We cannot be certain that the inclusion of different selection or a greater number of ECGs might have led to different results. For example, if more ECG samples had been selected with abnormalities in the ST segment, then there would have been more variability in the determination of the QRS duration. Therefore, the degree of differences we have observed within our examples might not be generalisable to other scenarios, but since the ECG samples were chosen to reflect daily clinical practice, it may in fact, be that these results might reflect realistic clinical situations quite appropriately.

Second, our results depend on the particular experts that were included in our study. We sought to reflect a real-life situation, so we did not only select cardiologists but a broad group of physicians who undertake some ECG analysis in their clinical practice. We cannot rule out that these results would have differed if only cardiologists or only intensive care physicians had been selected. Since patient management based on ECG findings is undertaken by many different specialties, we believe that our results would correctly reflect day-to-day clinical practice.

Third, one additional consideration regarding our analysis is the fact that there is no gold standard for the determination of the specific time points studied. Although there are preset rules that have been published, for any given ECG sample it can be difficult to reliably determine the actual onsets and ends of the specific ECG waveforms.^12^ Even computer-based algorithms do not overcome this shortcoming.^21^ Therefore, we did not focus on the mean duration of the intervals measured but only on the variability of the experts assessing these parameters. It was not our aim to find the ‘true’ values of the measured parameters but to determine whether there are essential differences in the assessments between differing physicians.

In summary, the determination of several time domain parameters of the ECG is not as objective as one might assume. The actual measurement depends on the performing physician and might therefore be prone to severe bias. This phenomenon can be clearly deduced from the high variability we have observed from our data. Whether this problem, which we believe is generally systematic, can be solved by computer algorithms has to be determined by further studies. Our results clearly demonstrate that any decision regarding invasive procedures or medications based solely upon determinations of time-domain ECG parameters might result in overtreatment or undertreatment.
